# Factors Associated with Compliance with Recommendations for Liver Cancer Screening in Korea: A Nationwide Survey in Korea

**DOI:** 10.1371/journal.pone.0068315

**Published:** 2013-06-28

**Authors:** Boyoung Park, Kui Son Choi, Mina Suh, Ji-Yeon Shin, Jae Kwan Jun

**Affiliations:** National Cancer Control Institute, National Cancer Center, Goyang-si, Korea; Sapporo Medical University, Japan

## Abstract

To investigate the factors associated with compliance with recommendations regarding liver cancer screening intervals and methods among individuals at high-risk for liver cancer in the Republic of Korea. We used data from the fourth Korean National Health and Nutrition Examination Survey (KNHANES IV), a representative cross-sectional nationwide survey conducted between 2007 and 2009. The liver cancer screening rate and factors associated with compliance with recommended screening intervals (6 months) and methods (both abdominal ultrasonography and serum alpha-fetoprotein testing) among individuals at high risk for liver cancer such as hepatitis B virus (HBV) carriers were investigated. Out of 24,871 KNHANES IV participants, 604 HBV carriers aged ≥20 years were included in our analysis. 39.6% of our study sample reported attending liver cancer screening at least once in their lifetime, 12.3% had attended within the previous 6 months, and 14.6% were screened using both recommended methods. Older age was associated with increased compliance with screening intervals (P-trend 0.011) and methods (40–49 year: OR = 3.25, 95% CI: 1.62–6.51; 50–59 years: OR = 3.09, 95% CI: 1.44–6.66; 60–69 years: OR = 3.17, 95% CI: 1.28–7.82). Unawareness of HBV infection status was negatively related to compliance with screening intervals and methods (OR = 0.30, 95% CI: 0.17–0.53; OR = 0.45, 95% CI: 0.26–0.79). Female sex (OR = 0.45, 95% CI: 0.25–0.78), lower household income (P-trend 0.011), and routine and manual occupations (OR = 0.46, 95% CI: 0.22–0.97) were associated with decreased compliance with screening methods. The liver cancer screening rate among high-risk individuals is much less suboptimal. Considering that those unaware of their HBV infection status got regular and complete liver cancer screening much less often, efforts should be made not only to decrease sociodemographic disparities, but also to better identify the high-risk population.

## Introduction

Liver cancer is the fifth most common cancer in men, the seventh most common in women, and the third most common cause of cancer death worldwide [1]. In the Republic of Korea, although the incidence and mortality of liver cancer have declined over the decades, it is still the fourth and sixth most common incident cancer in men and women, respectively, and the second most common cause of cancer death [2]. Chronic hepatitis B virus (HBV) infection and liver cirrhosis are the major risk factors for liver cancer [3], and chronic HBV infection is the most important risk factors for liver cirrhosis, especially in high-prevalence areas [4]. Korea is an endemic area of HBV infection. Although the prevalence of hepatitis B surface antigen (HBsAg) in Korea has decreased from 8.6% in 1980 to 3.2% in 2009 due to the nationwide HBV vaccination program implemented in 1995, Korea still shows intermediate endemicity [5].

Early detection of liver cancer through screening of individuals at high-risk for the disease is important for effective management [5]. However, surveillance programs for groups at high risk of developing liver cancer differ among countries [6]. In Western countries, such as Europe and the US, abdominal ultrasonography at 6-month intervals is recommended for liver cirrhosis patients, HBV carriers, or hepatitis C virus (HCV) carriers at high risk [7], [8]. Eastern countries, such as Japan and Korea, recommend abdominal ultrasonography combined with measurements of tumor markers, such as AFP, for groups at high risk of liver cancer [9], [10].

Previous studies have shown an association between socioeconomic, demographic factors and cancer screening attendance in gastric, breast, colorectal, and cervical cancer screening programs that target the general population [11]–[14]. However, factors associated with compliance with liver cancer screening intervals in a high-risk group have rarely been studied. Compliance with recommended screening methods has also been rarely investigated. Therefore, the aim of this study was to investigate the factors associated with compliance with recommended liver cancer screening intervals and methods in individuals at high-risk for liver cancer in Korea.

## Materials and Methods

### Study Population

Data from the fourth Korean National Health and Nutrition Examination Surveys (KNHANES IV) was used in this study. The KNHANES is a series of population based cross-sectional surveys that estimate the health and nutritional status of the Korean population. Four surveys have been completed thus far: I (1998), II (2001), III (2005), IV (2007–2009). A stratified multistage cluster probability sampling according to geographical area, age, and gender was applied to select representative samples of the non-institutionalized Korean population. KNHANES IV was composed of a health interview, a health behavior survey, a health examination survey, and a nutrition survey. Trained interviewers conducted a face-to-face interview at the participants’ household using a structured questionnaire. Details of the survey are fully described elsewhere [13], [14].

**Figure 1:**
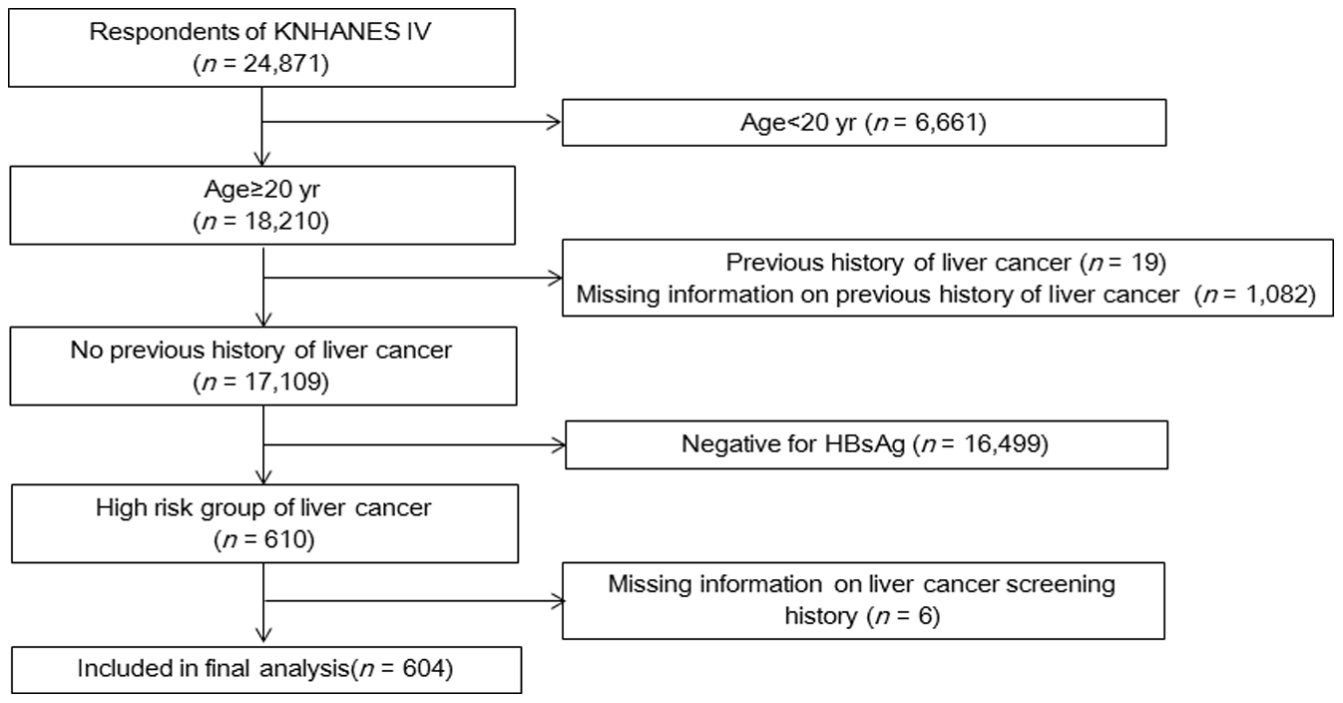
Flowchart of the study population. KNHANES IV (fourth Korean National Health and Nutrition Examination Surveys), HBsAg (hepatitis B surface antigen), and HCV(hepatitis C virus).

From the initial 31,705 individuals sampled for KNHANES IV, 24,871 participated in the survey (response rate: 78.4%). Among the 24,871 participants, we selected individuals aged 20 years or older without a known previous liver cancer history. Among the 17,109 individuals who met these criteria, 610 were at high risk for liver cancer with HBsAg-positive lab results. After excluding six individuals who did not answer questions on liver cancer screening attendance, 604 people were finally included in our analysis (Figure 1).

### Ethics Statement

The survey data are publicly available (http://knhanes.cdc.go.kr/knhanes/) and therefore ethics approval was not needed.

### Data Collection

KNHANES IV participants were asked whether they had ever been screened for liver cancer, if they were screened by abdominal ultrasonography and/or serum AFP test, and the time of their most recent liver cancer screening. We defined ‘lifetime liver cancer screening’ as having received liver cancer screening a minimum of one time in one’s lifetime, irrespective of the screening interval or method. ‘Regular liver cancer screening’ was defined as having received liver cancer screening within the previous 6 months. ‘Complete liver cancer screening’ was defined as being screened by both abdominal ultrasonography and serum AFP testing, and ‘incomplete liver cancer screening’ was defined as being tested by either abdominal ultrasonography or serum AFP testing alone, according to the recommendations of Eastern countries, such as Japan and Korea [9], [10].

HBsAg was measured in serum by electrochemiluminescence immunoassay. Demographic factors (sex, age, marital status, and residential area), socioeconomic factors (duration of education, monthly household income, occupation, insurance type, and adherence to private insurance), health-related factors (self-rated health status, self-rated depressive symptoms, and awareness of HBV infection status), and behavioral risk factors (alcohol drinking and smoking) were self-reported by participants. Awareness of HBV infection status was defined being HBsAg-positive and replying they had been previously diagnosed with HBV infection.

### Statistical Analysis

Basic characteristics of the study sample by lifetime liver cancer screening are presented as number and percentage, and were compared using the chi-square test. The lifetime liver cancer screening rates and regular liver cancer screening rates by screening method are presented as percentages by age group. The factors associated with regular and irregular liver cancer screening compared to never-screening were assessed using multivariate polychotomous logistic regression. To avoid over-adjustment by including an excessive number of variables [15], only variables with a statistical significance level less than 0.1 in univariate polychotomous logistic regression were included. The factors associated with complete and incomplete liver cancer screening compared to never-screening in the study population were also accessed using multivariate polychotomous logistic regression with the variables with a statistical significance level less than 0.1 in univariate analysis. All statistical analyses were performed using SAS software version 9.1 (SAS, Inc., Cary, NC, USA).

## Results

Among the 604 KNHANES IV participants without previous history of liver cancer and with HBsAg-positive included in our study sample, 297 (49.2%) were male and 307 (50.8%) were female. Distributions of age, marital status, monthly household income, self-rated health status, and awareness of HBV infection status were significantly different between those who reported lifetime liver cancer screening and those who never received liver cancer screening (Table 1).

**Table 1:** Basic characteristics of the study sample.

Variable	Lifetime liver cancer screening a		P-value
	Yes (N = 239)	No (N = 365)	
	N(%)	N(%)	
Demographic factors			
Sex			
Male	127(42.8)	170(57.2)	0.114
Female	112(36.5)	195(63.5)	
Age (years)			
<40	52(29.4)	125(70.6)	0.002
40−49	79(46.2)	92(53.8)	
50−59	60(48.8)	63(51.2)	
60−69	34(38.6)	54(61.4)	
≥70	14(31.1)	31(68.9)	
Marital status			
Married	197(41.5)	278(58.5)	0.032
Divorced/unmarried	39(30.9)	87(69.1)	
Residential area			
Urban	181(41.1)	259(58.9)	0.197
Rural	58(35.4)	106(64.6)	
Socioeconomic factors			
Duration of education (years)			
≤8	88(38.1)	143(61.9)	0.204
9−11	71(36.4)	124(63.6)	
≥12	80(44.9)	98(55.1)	
Monthly household income			
1st quartile	34(32.1)	72(67.9)	0.002
2nd quartile	48(32.9)	98(67.1)	
3rd quartile	54(36.7)	93(63.3)	
4th quartile	95(50.5)	93(49.5)	
Occupation			
Managerial and professional	49(49.5)	50(50.5)	0.151
Service and sales	27(35.1)	50(64.9)	
Routine and manual	74(37.6)	123(62.4)	
Unemployed/Housewives	87(38.0)	142(62.0)	
Insurance type			
National Health Insurance	229(39.6)	350(60.4)	0.682
Medical Aid Program	7(35.0)	13(65.0)	
Private insurance for health care			
Yes	172(40.9)	248(59.1)	0.329
No	63(36.6)	109(63.4)	
Health-related factors			
Self-rated health status			
Good	59(32.2)	124(67.8)	0.046
Moderate	107(41.8)	149(58.2)	
Poor	73(44.2)	92(55.8)	
Self –rated depressive symptoms			
Yes	39(38.6)	62(61.4)	0.830
No	200(39.8)	303(60.2)	
Awareness of HBV infection status			
Yes	74(52.9)	66(47.1)	<0.001
No	165(35.6)	299(64.4)	
Behavioral risk factors			
Alcohol drinking			
Current drinker	75(38.3)	121(61.7)	0.650
Former drinker/nondrinker	164(40.2)	244(59.8)	
Smoking			
Yes	130(37.0)	221(63.0)	0.124
No	109(43.3)	143(56.7)	

a Either ultrasonography or serum alpha-fetoprotein test.

**Figure 2:**
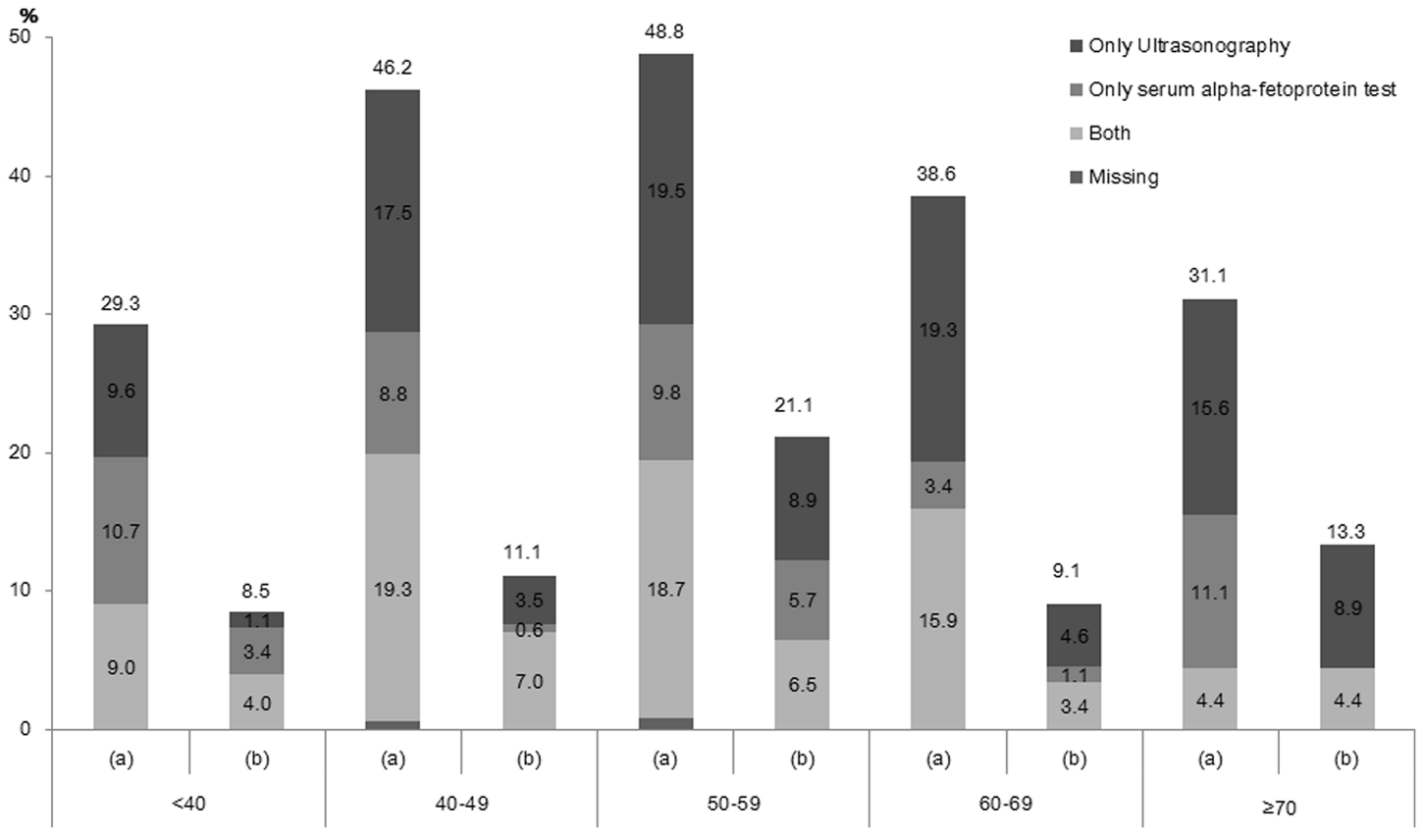
Lifetime (a) and regular (b) liver cancer screening rates according to screening method in high risk population of liver cancer by age. Lifetime liver cancer screening rate, regular liver cancer screening rate, and complete liver cancer screening in the entire study sample were 39.6%, 12.3%, and 14.6%, respectively.

Figure 2 presents the distributions of regularity and completeness of liver cancer screening by age group. Lifetime and regular liver cancer screening rates were highest in the age group 50–59 years (48.8% and 21.1%, respectively). Complete liver cancer screening was highest in the age group 40–49 years. Lifetime liver cancer screening rate, regular liver cancer screening rate, and complete liver cancer screening in the entire study sample were 39.6%, 12.3%, and 14.6%, respectively.

Table 2 shows the results of polychotomous logistic regression analyses for factors associated with regular and irregular liver cancer screening compared to never screening. Older age was associated with increased likelihood of regular liver cancer screening and marginally associated with irregular liver cancer screening (P-trend 0.011 and 0.061, respectively). Moderate self-reported health status was positively associated with regular liver cancer screening (OR = 2.15, 95% CI: 1.08–4.27) compared to good self-reported health status. Unawareness of HBV infection status was associated with lower regular liver cancer screening (OR = 0.30, 95% CI: 0.17–0.53). Lower monthly household income (P-trend 0.012) and working in the field of service and sales (OR = 0.41, 95% CI: 0.19–0.86), or in routine and manual occupations (OR = 0.53, 95% CI: 0.29–0.97) were associated with lower irregular liver cancer screening, and poor self-rated health status was related to increased irregular liver cancer screening (P-trend 0.011) (Table 2).

**Table 2:** Factors associated with regular (in previous 6 months) and irregular liver cancer screening compared to never-screening by polychotomous logistic regression.

Variable^a^	Regular liver cancer screening^b^		Irregular liver cancer screening^c^	
	aOR	(95% CI)	aOR	(95% CI)
Socio-demographic factors				
Age group (years)				
<40	1.00	(Ref)	1.00	(Ref)
40−49	1.82	(0.83–3.99)	2.69	(1.57–4.59)
50−59	3.69	(1.69–8.04)	2.34	(1.28–4.28)
60−69	1.98	(0.70–5.56)	2.45	(1.24–4.84)
≥70	3.08	(0.94–10.12)	1.30	(0.50–3.42)
P-trend	0.011		0.061	
Marital status				
Married	1.00	(Ref)	1.00	(Ref)
Divorced/unmarried	0.73	(0.35–1.51)	0.83	(0.50–1.39)
Monthly household income				
1st quartile	0.47	(0.19–1.17)	0.52	(0.27–0.99)
2nd quartile	0.48	(0.23–1.02)	0.48	(0.28–0.84)
3rd quartile	0.57	(0.28–1.19)	0.70	(0.41–1.18)
4th quartile	1.00	(Ref)	1.00	(Ref)
P-trend	0.057		0.012	
Occupation				
Managerial and professional	1.00	(Ref)	1.00	(Ref)
Service and sales	0.49	(0.18–1.30)	0.41	(0.19–0.86)
Routine and manual	0.55	(0.24–1.27)	0.53	(0.29–0.97)
Unemployed/Housewives	0.46	(0.21–1.04)	0.63	(0.35–1.13)
Health-related factors				
Self-reported health status				
Good	1.00	(Ref)	1.00	(Ref)
Moderate	2.15	(1.08–4.27)	1.57	(0.98–2.52)
Poor	1.99	(0.92–4.32)	1.99	(1.16–3.39)
P-trend	0.071		0.011	
Self –rated depressive symptoms				
Yes	1.43	(0.74–2.76)	0.60	(0.34–1.06)
No	1.00	(Ref)	1.00	(Ref)
Awareness of HBV infection status				
Yes	1.00	(Ref)	1.00	(Ref)
No	0.30	(0.17–0.53)	0.68	(0.43–1.09)

aOR: adjusted Odds Ratio, CI: Confidence Interval.

a Only variables with p-value<0.1 in the univariate analysis were included in multivariate analysis.

b Those who got liver cancer screening within previous 6 months.

c Those who got liver cancer screening but the interval was longer than 6 months.

Compared to never screening, lower household income was associated with both complete and incomplete liver cancer screening (P-trend 0.011 and 0.013, respectively). Being female, working in a routine and manual occupation, and unawareness of HBV infection status was associated with lower complete liver cancer screening (OR = 0.45, 95% CI: 0.25–0.78; OR = 0.46; 95% CI: 0.22–0.97; OR = 0.45, 95% CI: 0.26–0.79, respectively). Those aged 40–69 years got complete liver cancer screening more (age 40–49 years: OR = 3.25, 95% CI: 1.62–6.51; age 50–59: OR = 3.09, 95% CI: 1.44–6.66; age 60–69: OR = 3.17, 95% CI: 1.28–7.82 compared with under 40 years old). Older age and poorer self-reported health status were associated with higher incomplete liver cancer screening (P-trend 0.021 and 0.004, respectively). Working in service or sales occupations and being a former drinker/nondrinker were associated with lower incomplete liver cancer screening (OR = 0.37, 95% CI: 0.17–0.85 and OR = 0.60, 95% CI: 0.37–0.98, respectively) (Table 3).

**Table 3:** Factors associated with complete liver cancer screening (by both ultrasonography and serum alpha-fetoprotein testing) and incomplete screening compared to never-screening by polychotomous logistic regression.

Variable^a^	Complete liver cancer screening^b^		Incomplete liver cancer screening^c^	
	aOR	(95% CI)	aOR	(95% CI)
Socio-demographic factors				
Sex				
Male	1.00	(Ref)	1.00	(Ref)
Female	0.45	(0.25–0.78)	1.04	(0.67–1.61)
Age group (years)				
<40	1.00	(Ref)	1.00	(Ref)
40–49	3.25	(1.62–6.51)	2.20	(1.27–3.81)
50–59	3.09	(1.44–6.66)	2.62	(1.43–4.79)
60–69	3.17	(1.28–7.82)	2.11	(1.01–4.38)
70–	0.67	(0.13–3.48)	2.31	(0.92–5.76)
P-trend	0.163		0.016	
Monthly household income				
1st quartile	0.28	(0.11–0.73)	0.60	(0.31–1.14)
2nd quartile	0.59	(0.30–1.14)	0.41	(0.23–0.73)
3rd quartile	0.67	(0.35–1.30)	0.60	(0.35–1.04)
4th quartile	1.00	(Ref)	1.00	(Ref)
P-trend	0.011		0.013	
Occupation				
Managerial and professional	1.00	(Ref)	1.00	(Ref)
Service and sales	0.58	(0.24–1.41)	0.37	(0.17–0.85)
Routine and manual	0.46	(0.22–0.97)	0.64	(0.34–1.21)
Unemployed/Housewives	0.58	(0.27–1.26)	0.69	(0.37–1.29)
Health-related factors				
Self-reported health status				
Good	1.00	(Ref)	1.00	(Ref)
Moderate	1.03	(0.56–1.87)	2.45	(1.48–4.07)
Poor	1.64	(0.85–3.19)	2.23	(1.25–3.97)
P-trend	0.149		0.004	
Awareness of HBV infection status				
Yes	1.00	(Ref)	1.00	(Ref)
No	0.45	(0.26–0.79)	0.58	(0.36–0.94)
Alcohol drinking				
Current drinker	1.00	(Ref)	1.00	(Ref)
Former drinker/nondrinker	1.40	(0.80–2.46)	0.60	(0.37–0.98)

aOR: adjusted Odds Ratio, CI: Confidence Interval.

a Only variables with p-value<0.1 in the univariate analysis were included in multivariate analysis.

b Both ultrasonography and serum alpha-fetoprotein test.

c Either ultrasonography or serum alpha-fetoprotein test.

## Discussion

Considering the disease burden of liver cancer and its low survival rate [2], early diagnosis of liver cancer through screening is important in reducing the corresponding cost and mortality. The results of the present study showed that only 39.6% of our study sample, made up of individuals at high risk for liver cancer aged 20 or more ever got liver cancer screening in their lifetime, 12.3% reported regular screening (i.e., screening in the previous 6 months), and 14.6% reported complete screening (i.e., screening by both abdominal ultrasonography and serum AFP testing). We found that older age, moderate self-reported health status, and awareness of HBV infection status were associated with increased regular liver cancer screening rate, compared with never-screening. Being female, having a lower household income, working in a routine or manual occupation, and unawareness of HBV infection status were associated with decreased complete screening rate, and people aged 40–69 years had higher complete liver cancer rate.

Previous studies conducted in Korea showed that the lifetime liver cancer screening rate among individuals at high risk for the disease increased from 31.8% in 2004 to 54.3% in 2011 [16]. These studies targeted high-risk individuals such as chronic HBV or HCV carriers, or liver cirrhosis patients aged 40 years or more. When we restricted our analyses to those aged 40 or more, the lifetime liver cancer screening rate was 43.8% (data not shown). These results including our study were lower than the results of other countries [17], [18]. Although receiving liver cancer screening every 6 months for high-risk individuals have been recommended considering tumor doubling time [10], only 12.3% of our study sample reported regular liver cancer screening. Our results fell between those of the study conducted by Noh et al., which reported a 6% liver cancer screening rate within the previous 6 months [10], and the 22.9% in the study by Park et al [16]. However, all of these results are much lower than those from China in which 23.5% or 40.6% got regular screening [19].

Older age was associated with both increased regular and complete liver cancer screening. In Korea, the National Cancer Screening Program, an organized screening program for gastric, liver, colorectal, breast, and cervical cancers provided by government, provided liver cancer screening using both abdominal ultrasonography and serum AFP testing every 6 months for those aged 40 years old or over who are HBsAg-positive, anti-HCV positive, or have liver cirrhosis either for free, or with a 10% deductible [10], [20]. This organized screening program may contribute to the higher regular and complete screening rates we observed in the older age groups compared with those less than 40 years old.

A previous study showed that HBV or HCV carriers who were aware of their infection status got abdominal ultrasonography more often during their lifetime [21], whereas in our study they received regular and complete liver cancer screening more often. Perceived risk has been positively associated with cancer screening [22] and therefore, it could be suggested that large a proportion of high risk group did not get appropriate liver cancer screening because they did not know that they were in high risk of liver cancer. In our study 77% of HBV carriers (464 of 604) were unaware of their infection status. This is much lower than a previous study in Korea, which reported that only one-fourth of HBV carriers were unaware of their infection status [23]. However, that study population was composed of participants of an opportunistic cancer screening program conducted on the basis of personal preference in a single general hospital and they were considered to be concerned about their health status more.

The relationship between self-rated health status and cancer screening is controversial. Some have reported that a better self-rated health status was associated with increased cancer screening attendance [24], [25], while others suggested that those with better self-rated health status were less likely to attend cancer screening [26]–[28]. In our results, moderate self-reported health status was associated with increased regular liver cancer screening but poor self-rated health status did not show significant results compared with good self-rated health status. However, the trend showed marginal significance showing that poorer self-rated health status increased the regular screening rate (P-trend 0.071). However, poorer self-rated health status increased the irregular and incomplete screening rate compared to never-screening.

Compared with males, females were less likely to get complete liver cancer screening. One possible explanation could be that males have more opportunities to attend organization- and government-supported screening in the workplace. Our results regarding the association between household income and complete liver cancer screening were in accordance with those from previous studies on screening behavior for other cancer types in general population. Higher household income was associated with a higher cancer screening rate for colorectal, gastric, breast, and cervical cancer screening [29]. Occupation reflected socioeconomic status in our study, and affected the complete liver cancer screening rate.

This study has several limitations. First, as all the information was measured by self-report in health surveys, therefore we cannot rule out the possibility of information bias. Indeed, self-reported information on liver cancer screening and screening method was not matched to medical records. However, previous studies have shown that self-reported cancer screening history is reliable, and agrees well with medical records [30], [31]. Second, we restricted high-risk individuals to those with positive serum assays for HBsAg because we did not measure HCV antibodies in serum, although people who were anti-HCV-positive were included in the target population of the National Cancer Screening Program in Korea. Considering that the target population of the National Cancer Screening Program in Korea included 4.6% of anti-HCV carriers, the study population might not represent the entire high-risk group for liver cancer, although it was representative of total HBV carriers.

Despite of these limitations, this study has several strengths. The results are based on a large, nationally representative sample of high-risk individuals. Second, to our knowledge, this is the first study to investigate factors associated with regularity and completeness of liver cancer screening in individuals at high risk for liver cancer and we expect that the results might provide new opportunities to explore liver cancer screening behavior and practice in this population.

As Korea remains an area of intermediate HBV prevalence, and that the burden of liver cancer is especially great among individuals with low socioeconomic status [32], efforts to increase the liver cancer screening rate, following recommended methods (both abdominal ultrasonography and serum AFP testing) and intervals (6 months) for those at high risk are necessary. In conclusion, our finding indicate that the lifetime screening rate, regular screening rate, and complete screening rate for liver cancer were much less suboptimal, and age and awareness of HBV infection status were positively associated with both regular and complete liver cancer screening in our study sample of individuals at high risk for liver cancer. In addition, other sociodemographic factors such as sex, household income, and occupation were associated with regular and complete liver cancer screening. The strategies for increasing liver cancer screening should be different from those for other cancers, which target the general population. Considering that those unaware of their HBV infection status got regular and complete liver cancer screening much less often, efforts should be made not only to decrease sociodemographic disparities, but also to better identify the high-risk population. Although the National Cancer Screening Program for liver cancer offers HBsAg and anti-HCV testing for free to Medical Aid Program recipients, the participation rates were low. Therefore, increasing the awareness of guidelines for liver cancer screening to large proportion of population in Korea might be needed. Further studies on the enhancement of liver cancer screening rate targeting high risk group are warranted.

## References

[1] Ferlay J, Shin HR, Bray F, Forman D, Mathers C, et al (2010) Estimates of worldwide burden of cancer in 2008: GLOBOCAN 2008. Int J Cancer 127: 2893–2917. 21351269 10.1002/ijc.25516

[2] Jung KW, Park S, Kong HJ, Won YJ, Lee JY, et al (2012) Cancer statistics in Korea: incidence, mortality, survival, and prevalence in 2009. Cancer Res Treat 44: 11–24. 22500156 10.4143/crt.2012.44.1.11 PMC3322196

[3] El-Serag HB (2002) Hepatocellular carcinoma: an epidemiologic view. J Clin Gastroenterol 35: S72–78. 12394209 10.1097/00004836-200211002-00002

[4] Kim YS, Um SH, Ryu HS, Lee JB, Lee JW, et al (2003) The prognosis of liver cirrhosis in recent years in Korea. J Korean Med Sci 18: 833–841. 14676440 10.3346/jkms.2003.18.6.833 PMC3055146

[5] Park B, Choi KS, Lee HY, Jun JK, Park EC (2012) Socioeconomic inequalities in completion of hepatitis B vaccine series among Korean women: results from a nationwide interview survey. Vaccine 30: 5844–5848. 22828587 10.1016/j.vaccine.2012.07.022

[6] Kim do Y, Kim JW, Kuromatsu R, Ahn SH, Torimura T, et al (2011) Controversies in surveillance and early diagnosis of hepatocellular carcinoma. Oncology 81 Suppl 156–60. 22212937 10.1159/000333261

[7] European Association For The Study Of The Liver; European Organisation For Research And Treatment Of Cancer (2012) EASL-EORTC clinical practice guidelines: management of hepatocellular carcinoma. J Hepatol 56: 908–943. 22424438 10.1016/j.jhep.2011.12.001

[8] Bruix J, Sherman M (2011) Management of hepatocellular carcinoma: an update. Hepatology 53: 1020–1022. 21374666 10.1002/hep.24199 PMC3084991

[9] Kudo M, Izumi N, Kokudo N, Matsui O, Sakamoto M, et al (2011) Management of hepatocellular carcinoma in Japan: Consensus-Based Clinical Practice Guidelines proposed by the Japan Society of Hepatology (JSH) 2010 updated version. Dig Dis 29: 339–364. 21829027 10.1159/000327577

[10] Noh DK, Choi KS, Jun JK, Lee HY, Park EC (2012) Factors associated with attending the National Cancer Screening Program for liver cancer in Korea. Asian Pac J Cancer Prev 13: 731–736. 22524852 10.7314/apjcp.2012.13.2.731

[11] Lee HY, Park EC, Jun JK, Hahm MI, Jung KW, et al (2010) Trends in socioeconomic disparities in organized and opportunistic gastric cancer screening in Korea (2005–2009). Cancer Epidemiol Biomarkers Prev 19: 1919–1926. 20647409 10.1158/1055-9965.EPI-09-1308

[12] Park MJ, Park EC, Choi KS, Jun JK, Lee HY (2011) Sociodemographic gradients in breast and cervical cancer screening in Korea: the Korean National Cancer Screening Survey (KNCSS) 2005–2009. BMC Cancer 11: 257. 21682886 10.1186/1471-2407-11-257 PMC3144456

[13] Kwon YM, Lim HT, Lee K, Cho BL, Park MS, et al (2009) Factors associated with use of gastric cancer screening services in Korea. World J Gastroenterol 15: 3653–3659. 19653344 10.3748/wjg.15.3653 PMC2721240

[14] Myong JP, Shin JY, Kim SJ (2012) Factors associated with participation in colorectal cancer screening in Korea: the Fourth Korean National Health and Nutrition Examination Survey (KNHANES IV). Int J Colorectal Dis 27: 1061–1069. 22354136 10.1007/s00384-012-1428-4

[15] Breslow N (1982) Design and analysis of case-control studies. Annu Rev Public Health 3: 29–54. 6756431 10.1146/annurev.pu.03.050182.000333

[16] Park B, Choi KS, Lee YY, Jun JK, Seo HG (2012) Trends in Cancer Screening Rates among Korean Men and Women: Results from the Korean National Cancer Screening Survey (KNCSS), 2004–2011. Cancer Res Treat 44: 113–120. 22802749 10.4143/crt.2012.44.2.113 PMC3394860

[17] Zhang BH, Yang BH, Tang ZY (2004) Randomized controlled trial of screening for hepatocellular carcinoma. J Cancer Res Clin Oncol 130: 417–422. 15042359 10.1007/s00432-004-0552-0 PMC12161851

[18] McMahon BJ, Bulkow L, Harpster A, Snowball M, Lanier A, et al (2000) Screening for hepatocellular carcinoma in Alaska natives infected with chronic hepatitis B: a 16-year population-based study. Hepatology 32: 842–846. 11003632 10.1053/jhep.2000.17914

[19] Chen JG, Parkin DM, Chen QG, Lu JH, Shen QJ, et al (2003) Screening for liver cancer: results of a randomised controlled trial in Qidong, China. J Med Screen 10: 204–209. 14738659 10.1258/096914103771773320

[20] Lee EH, Han MA, Lee HY, Jun JK, Choi KS, et al (2010) Liver cancer screening in Korea: a report on the 2008 National Cancer Screening Programme. Asian Pac J Cancer Prev 11: 1305–1310. 21198282

[21] Cho ER, Shin A, Choi KS, Lee HY, Kim J (2010) Factors associated with use of ultrasonography screening for hepatocellular carcinoma among hepatitis B or C carriers. Cancer Epidemiol 34: 713–716. 20947465 10.1016/j.canep.2010.09.003

[22] Vernon SW (1999) Risk perception and risk communication for cancer screening behaviors: a review. J Natl Cancer Inst Monogr: 101–119. 10.1093/oxfordjournals.jncimonographs.a024184 10854465

[23] Shin A, Cho ER, Kim J, Sung J, Park KW, et al (2009) Factors associated with awareness of infection status among chronic hepatitis B and C carriers in Korea. Cancer Epidemiol Biomarkers Prev 18: 1894–1898. 19454614 10.1158/1055-9965.EPI-08-1228

[24] Burack RC, Gurney JG, McDaniel AM (1998) Health status and mammography use among older women. J Gen Intern Med 13: 366–372. 9669565 10.1046/j.1525-1497.1998.00116.x PMC1496965

[25] Miles A, Rainbow S, von Wagner C (2011) Cancer fatalism and poor self-rated health mediate the association between socioeconomic status and uptake of colorectal cancer screening in England. Cancer Epidemiol Biomarkers Prev 20: 2132–2140. 21953115 10.1158/1055-9965.EPI-11-0453 PMC3199581

[26] Carlos RC, Fendrick AM, Patterson SK, Bernstein SJ (2005) Associations in breast and colon cancer screening behavior in women. Acad Radiol 12: 451–458. 15831418 10.1016/j.acra.2004.12.024

[27] Selvin E, Brett KM (2003) Breast and cervical cancer screening: sociodemographic predictors among White, Black, and Hispanic women. Am J Public Health 93: 618–623. 12660207 10.2105/ajph.93.4.618 PMC1447800

[28] Jerant AF, Franks P, Jackson JE, Doescher MP (2004) Age-related disparities in cancer screening: analysis of 2001 Behavioral Risk Factor Surveillance System data. Ann Fam Med 2: 481–487. 15506585 10.1370/afm.118 PMC1466727

[29] Park B, Choi KS, Lee YY, Jun JK, Seo HG (2012) Cancer screening status in Korea, 2011: results from the Korean National Cancer Screening Survey. Asian Pac J Cancer Prev 13: 1187–1191. 22799303 10.7314/apjcp.2012.13.4.1187

[30] Caplan LS, McQueen DV, Qualters JR, Leff M, Garrett C, et al (2003) Validity of women’s self-reports of cancer screening test utilization in a managed care population. Cancer Epidemiol Biomarkers Prev 12: 1182–1187. 14652278

[31] Khoja S, McGregor SE, Hilsden RJ (2007) Validation of self-reported history of colorectal cancer screening. Can Fam Physician 53: 1192–1197. 17872816 PMC1949303

[32] Khang YH, Lynch JW, Kaplan GA (2004) Health inequalities in Korea: age- and sex-specific educational differences in the 10 leading causes of death. Int J Epidemiol 33: 299–308. 15082630 10.1093/ije/dyg244

